# Inflammatory parameters and prediction of prognosis in infective endocarditis

**DOI:** 10.1186/1471-2334-13-272

**Published:** 2013-06-15

**Authors:** Christian G Cornelissen, Dirk A Frechen, Karin Schreiner, Nikolaus Marx, Stefan Krüger

**Affiliations:** 1 Medizinische Klinik I, Universitätsklinikum RWTH Aachen, Pauwelsstraße 30, Aachen, 52074, Germany

## Abstract

**Background:**

Procalcitonin (PCT) is widely used in critically ill patients to diagnose clinically significant infection and sepsis. Aim of this study was to evaluate the prognostic value of PCT in comparison to white blood cell count (WBC) and C-reactive protein (CRP) for clinical outcome and its correlation with microbiological etiology in patients with infective endocarditis (IE).

**Methods:**

A retrospective single-center analysis was performed from 2007 till 2009. All patients were diagnosed having IE according to Duke standard criteria. Before starting antibiotic therapy, WBC, CRP and PCT were measured and blood cultures were taken for microbiological diagnosis of the etiological pathogen. Patients were followed up during in-hospital stay for poor outcome, defined as death or serious complications due to IE.

**Results:**

During the study period 50 patients (57 ± 17 years, 72% male) fulfilling Duke criteria for IE were identified. In all patients PCT measurements before start of antibiotic therapy were available. In ROC analysis, a cut-off for PCT > 0.5 ng/mL was most accurate for the prediction of poor outcome with a sensitivity of 73% and specificity of 79%, a positive predictive value of 79% and a negative predictive value of 73%. Patients with a PCT > 0.5 ng/mL had an odds ratio of 12.8 (95% CI 2.5 – 66.2) for finding *Staphylococcus aureus* in blood cultures.

**Conclusions:**

For the first time, this study shows that in IE, an initial value of PCT > 0.5 ng/mL is a useful predictor of poor outcome, i.e. death or serious infectious complications. PCT > 0.5 ng/mL should raise the suspicion of *Staphylococcus aureus* as the etiological pathogen, whereas PCT levels < 0.5 ng/mL make staphylococcal infection unlikely.

## Background

The term “infective endocarditis” (IE) is used to describe a set of clinically different entities. The morbidity and mortality of IE remains high. Right sided native valve IE generally takes a more benign course and even short-term antibiotic regimen can be successful [1]. Prosthetic valve IE, by contrast, is a severe, life-threatening disease requiring different therapeutic measures [2]. In IE, known predictors of clinical outcome are age, vegetation size and the causative organism [3-7]. Still, individual clinical courses differ significantly.

Thus, a biomarker for the prediction of prognosis and the identification of the etiological pathogen at the initial evaluation of patients with IE would be very valuable and helpful. A biomarker strategy could allow early identification of high-risk IE patients needing more aggressive therapy. Up to now, C-reactive protein (CRP) has been studied as a predictor of clinical course in IE. Serial measurements showing elevated serum CRP levels > 122 mg / dl one [8] and > 62 mg / dl four [9] weeks after initiation of treatment have shown to predict poor outcome, but initial serum levels of CRP at time of diagnosis failed to predict the clinical course [8-10].

Procalcitonin (PCT) is widely used in critically ill patients to diagnose clinical significant infection and sepsis. In IE patients undergoing heart valve replacement, PCT showed typical postoperative kinetics with a peak 3 days after surgery but failed to predict complications of surgery [11]*.* It has also been found to be a valuable diagnostic marker in IE [12,13], but its prognostic value has not yet been investigated.

The aim of this study was therefore to evaluate the prognostic value of PCT for clinical outcome including death and serious complications and its correlation with microbiological etiology in patients with IE.

## Methods

### Patients

We performed a retrospective single-centre study at a German university hospital with large departments of cardiology and cardiac surgery. Data from cardiologic patients were analysed from January 1st 2007 until December 31st 2009 in accordance with the Helsinki declaration. Written approval was obtained from the ethics committee of the RWTH Aachen university hospital for this study. All patients with documented diagnosis of IE were included into the study. Clinical documentation was evaluated for the presence of Duke endocarditis service criteria [14]. Patients that did not match Duke criteria for definite IE were excluded from the analysis. All patients that were positive for definite IE according to the Durack criteria also fulfilled the modified Duke criteria for definite IE [15].

In house medical records for eligible patients were obtained. All clinical relevant data from the patients were stored in an electronic database. Data set included patients’ characteristics, laboratory measurements, echocardiography, microbiological findings, pathological findings, need for surgical valve replacement of the infected valve and clinical course of the disease. Detection of microbial pathogens was performed according to standard methods and established microbiological guidelines. All patients were followed-up until demission from hospital.

During the study period 67 patients with the diagnosis of IE were treated at our hospital. In the retrospective analysis nine patients failed to match Duke endocarditis service’s criteria for IE. In patients fulfilling the Duke criteria, eight had no initial PCT measurement before start of antibiotic therapy and were therefore excluded from the study. In total, 50 patients qualified for further analysis.

### Determination of PCT, CRP and leukocyte count

Leukocyte count (WBC) was determined by the hospital laboratory. Serum CRP *(normal < 5 mg/dl)* was measured by nephelometry with a commercially available assay (Behring Diagnostics, Marburg, Germany). Serum PCT *(normal < 0.1 pg/nl)* was determined by an immunofluorescent assay (B.R.A.H.M.S PCT sensitive KRYPTOR, B.R.A.H.M.S AG, Henningsdorf, Germany). The assay requires 50 μl of serum, EDTA or heparin plasma, has a functional assay sensitivity (defined as lowest value with an interassay CV <20%) of 0.06 ng/mL and a lower detection limit of 0.02 ng/mL. Laboratory measurements were performed in a blinded fashion without knowledge of the microbiological results or the clinical status of the patient. PCT, CRP and WBC were measured on admission before initiation of antibiotic therapy. Patients without measurement of PCT, CRP and WBC before initiation of antibiotic therapy were excluded from the study.

### Clinical outcome

The primary end point of this study was poor clinical outcome, which was defined as either death or serious complications during hospitalisation. Serious complications included infectious complications (defined as septic arthritis, meningitis, osteomyelitis, visceral abscess, peripheral emboli, mycotic aneurysm, septic pulmonary infarction and intracranial hemorrhage or infarction), cardiac failure, severe cardiac rhythm disorders, septic shock and need for surgical replacement of the infected valve. This combined endpoint was also used in previous larger studies in this field - e.g. by Verhagen et al. [8].

### Statistics

Continuous variables are expressed as mean ± SD or median and interquartile range in parenthesis unless stated otherwise. Two group nonparametric comparisons were calculated by the Mann–Whitney U-test. Frequency comparison was done using the χ2-test. To compare the predictive value of WBC, CRP and PCT we constructed Receiver-operating-characteristics (ROC) curves and determined the area under the curve (AUC). The outcome variable was event-free survival (no death, no serious complication) until demission from hospital. The operative characteristics of PCT, CRP and WBC were assessed calculating sensitivity, specificity, positive and negative predictive values and the positive and negative likelihood-ratio. Kaplan Meier survival curves were generated to visualize the distribution of times from baseline to death or serious complications, and log-rank test was performed to compare the event-free survival curves between groups. All statistical tests were 2-tailed and a p-value < 0.05 was considered statistically significant. Data analysis was performed using commercially available software (SAS enterprise guide version 4, SAS Institute Inc., NC, USA).

## Results

The study population had a mean age of 57 ± 17 years (range 23 to 87 years), and 72% were male (Table 1). All patients were hospitalised. In most cases the aortic (58%) or mitral valve (28%) were involved. The mortality rate of the study population was 22% (n = 11). At follow-up 18 patients suffered from serious complications (for details please see Table 2). According to international guidelines, appropriate antibiotic treatment was initiated as early as possible in all patients with IE or high probability of IE. Empiric antibiotic therapy was administered as a combination therapy and pathogen-specific therapy was initiated as soon as antibiograms were available. The mean length of stay in hospital was 29 ± 17 days (range 3 to 65 days) for patients with event-free survival and 38 ± 28 days (range 1 to 120 days) for patients with adverse events.

**Table 1:** Patients characteristics

**Characteristic**	**Total** **(n = 50)**	**Adverse event** **(n = 27)**	**Event-free survival** **(n = 23)**	**p-value**
**Age**, (years ± SD)	57 ± 17	52 ± 16	62 ± 16	
**Male / Female** (n)	36 / 14	18 / 9	18 / 5	
**Charlson Comorbidity Score** age adjusted				
(Median, interquartile range)	1 [0 – 4]	1 [0 – 4]	0 [0 – 4,5]	0.89
**Diabetes Mellitus** (n)	8	6	2	0.19
Immunocompromised* (n)	1	1	0	--
**i.v. Drug Abuse** (n)	6	6	0	0.02
**Conditions at diagnosis** (n)				
Vasopressors	15	12	3	0.02
Mechanical ventilation	11	8	3	0.16
ICU	17	12	5	0.09
**Site of infection** (n, %)				
Aortic valve	29 (58%)	13	16	0.58
Pulmonary valve	1 (2%)	1	0	--
Mitral valve	14 (28%)	8	6	0.59
Tricuspid valve	6 (12%)	5	1	--
**Prosthetic valve** (n)	11	8	3	0.16
**Known valvular disease** (n)	13	7	6	1
**Echocardiography**				
Perivalvular abscedation (n)	5	4	1	0.22
Severe Insufficiency (n)	21	8	13	0.05
Size of vegetation				
(2D, mm2, median, interquartile range)	103 [32 – 179]	105 [54 – 195]	98 [27 – 162]	0.22
**Causative organism** (n, %)				
*Staphylococcus aureus*	16 (32%)	15	1	< 0.01
*Staphylococci other than aureus*	7 (14%)	2	0	--
*B-Streptococci*	2 (4%)	0	2	--
*Streptococci other than B*	11 (22%)	2	9	0.03
*Enterococcus faecium*	5 (10%)	2	3	--
*Actinomyces israelii*	1 (2%)	0	1	--
*No proven causative organism*	7 (14%)	4	0	--

Data are presented as number of cases (percentages of total number).

*Patients with human immunodeficiency virus, acquired immunodeficiency syndrome, solid organ or bone marrow transplantation, receiving steroids (>20 mg of prednisone or equivalent for >1 month), or recent chemotherapy (>1 month).

**Table 2:** Clinicial outcome and complications

**Adverse outcome**	**27 (54%)**
**Death**	11 (22%)
**Serious infectious complication**	18 (36%)
meningitis	6 (12%)
peripheral embolisation	7 (14%)
visceral abscess	4 (8%)
osteomyelitis	3 (6%)
**Other serious complication**	7 (14%)
cardiac failure	2 (4%)
severe cardiac rhythm disorders	0 (0%)
septic shock	5 (10%)
**Need for valve replacement**	29 (58%)
(aortic n = 19, mitral n = 7, pulmonary n = 1, tricuspid n = 2)	

Data are presented as number of cases (percentages of total number).

### Prediction of death or serious complications from IE

**Figure 1:**
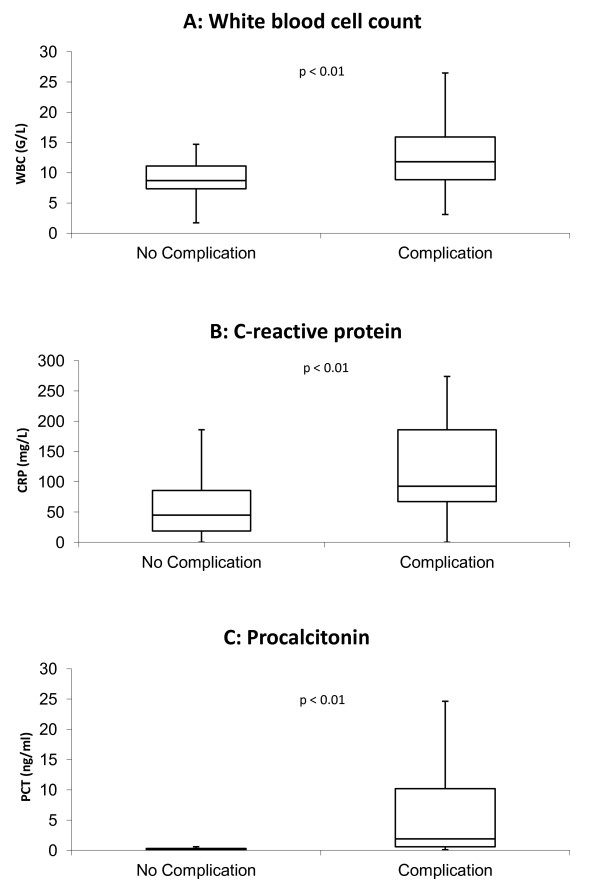
White blood cell count, C-reactive protein and Procalcitonin levels for patients with and without serious complications.

**Figure 2:**
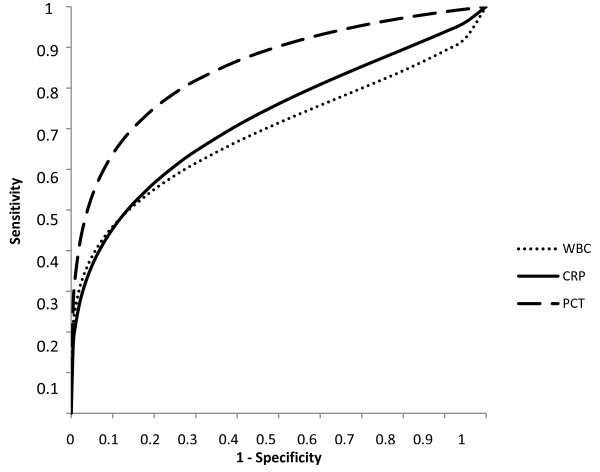
Receiver operating curves for white blood cell count, C-reactive protein and Procalcitonin for the prediction of adverse events.

**Figure 3:**
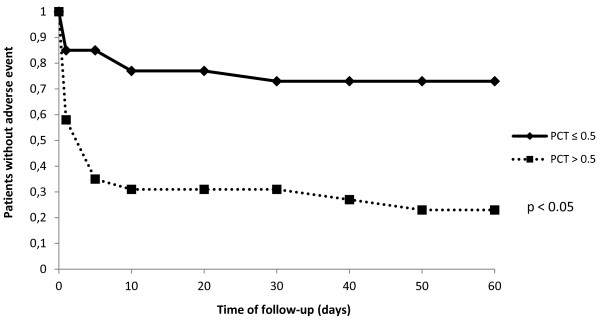
Kaplan-Meier event-free survival curves for a PCT > 0.5.

Median PCT levels (Table 3) on admission of patients with death or serious complications were significantly higher compared to those in event-free survivors (1.7 [0.6 – 8.6] vs. 0.2 [0.1 – 0.3] ng/mL, p < 0.01, Figure 1a). The respective values for CRP were 93 [63 – 193] vs. 45 [19 – 86] mg/L, p < 0.01 (Figure 1b) and for WBC 11.6 [7.9 – 16.0] vs. 8.7 [7.4 – 11.1] G/L, p < 0.01 (Figure 1c). The accuracy of WBC, CRP and PCT to predict death or serious complications at follow-up according to receiver operating characteristics is given in Figure 2. The AUC was highest for PCT (0.85 ± 0.05), which was significantly higher compared to the AUC for CRP (0.73 ± 0.07, p < 0.01) and WBC (0.69 ± 0.07, p < 0.01). In ROC-analysis, a PCT cut-off > 0.5 ng/mL was found to be the best predictor of adverse outcome with a sensitivity of 73%, specificity of 79%, positive predictive value of 79% and negative predictive value of 73%. Figure 3 shows Kaplan Meier survival curves of low risk patients and high risk patients stratified according to a PCT cut-off level of 0.5 ng/mL. Mortality and serious complications were significantly lower in patients with a PCT ≤ 0.5 ng/mL compared to patients with PCT values > 0.5 ng/mL.

**Table 3:** Median (interquartile range) levels of biomarkers

**Characteristic**	**Adverse event** **(n = 26)**	**Event-free survival** **(n = 24)**	**p-value**	***Staph. aureus*IE** **(n = 16)**	***Non Staph. aureus*IE** **(n = 14)**	**p-value**
**Procalcitonin**	1.7	0.2	< 0.01	2.1	0.5	0.36
(ng/mL)	[0.6 – 8.6]	[0.1 – 0.3]		[0.9 – 10.2]	[0.2 – 2.1]	
**CRP**	93	86	< 0.01	169	86	0.11
(mg/L)	[63–193]	[19–86]		[48–230]	[33 – 163]	
**WBC**	11.6	8.7	<0.01	11.7	10.1	0.28
(G/L)	[7.9 – 16.0]	[7.4 – 11.1]		[10.6 – 15.9]	[7.2 – 13.4]	

Legend: IE: infective endocarditis; CRP: C-reactive protein; WBC: white blood cell count.

### Microbiology and inflammatory markers

**Figure 4:**
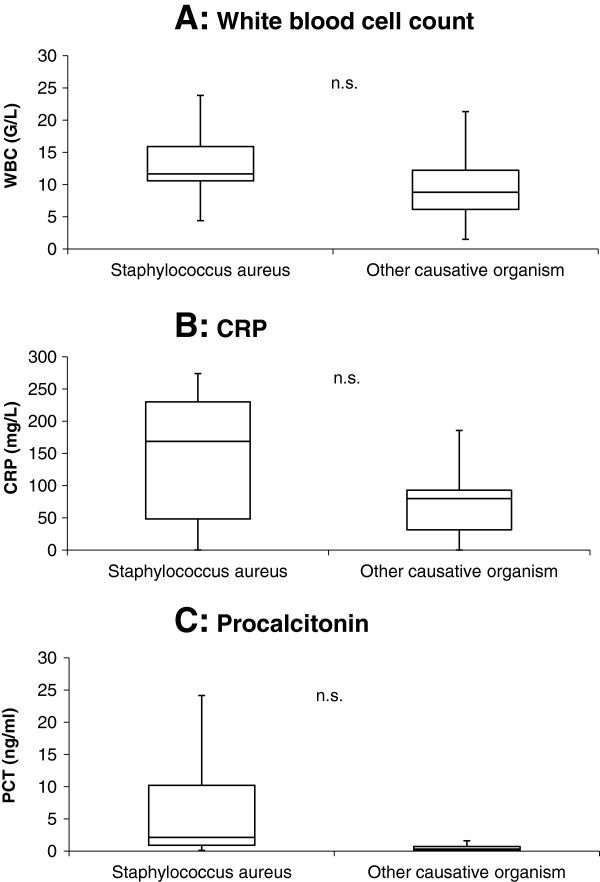
White blood cell count, C-reactive protein and Procalcitonin levels for Staph. aureus and other causative organisms.

The causative pathogen was found in 43 (86%) patients (Table 1). Patients with IE due to an infection with Staphylococcus aureus compared to other pathogens (Figure 4) showed no significant differences in values of PCT (2.1 [0.9 – 2.1] vs. 0.5 [0.2 – 2.1] ng/mL, p = 0.36), CRP (169 [48 – 230] vs. 86 [33 – 163] mg/L, p = 0.11) and WBC (11.7 [10.6 – 15.9] vs. 10.1 [7.2 – 13.4] G/L, p = 0.28). At univariate analysis, serum PCT > 0.5 ng /mL showed an odds ratio of 12.8 (95% CI 2.5 to 66.2) to differentiate IE due to Staphylococcus aureus from IE due to other etiology.

## Discussion

The main findings of our study include 1) that levels of PCT, CRP and WBC are significantly higher in patients that experience death or serious complications in IE, 2) PCT is the best predictor of poor clinical outcome, 3) initial biomarker measurement does not allow individual prediction of microbiological etiology of IE.

The current diagnostic strategy for IE follows the Duke criteria [14,15]. It requires typical microorganisms grown from at least 2 separate blood cultures and evidence of endocardial involvement, usually documented by echocardiography. Despite these criteria the definitive diagnosis of IE in daily clinical practice requires expertise and time. Therefore, it would be very helpful if we had a biomarker for the rapid evaluation of the presence or absence of IE in suspected cases.

There are some previous studies with respect to IE and inflammatory biomarkers. All have in common that the biomarkers, especially PCT, are used for the diagnosis or differential diagnosis of IE. Müller et al. conducted a prospective cohort study in 67 consecutive patients with the suspicion of IE [13]. Comparable to our study, the diagnosis of IE was established by application of the Duke criteria. In 21 patients with confirmed IE, PCT was significantly higher in patients with IE (median 6.56 ng/mL) than in those with other final diagnoses (median 0.44 ng/mL). The AUC for PCT (0.856) to predict IE was significantly higher compared to CRP (0.657). Interestingly, PCT was the only significant independent predictor of IE on admission in multivariate analysis. Another study by Knudsen et al. found similar results [16]. PCT was higher in patients with IE compared to those where IE diagnosis was rejected. In this study independent determinants of high PCT were positive blood culture with endocarditis-typical microorganisms, temperature ≥38°C, symptoms ≤ 5 days, immunocompromised status and male gender. However, the range of PCT was that wide, that a suitable PCT threshold for diagnosing or excluding IE could not be established in this study.

Our study is the first to evaluate inflammatory biomarkers at initial presentation for the prediction of prognosis of IE in terms of death and serious complications, which has not been addressed in previous studies. Previous studies included either the analysis of a variety of risk factors [17] or serial measurements of CRP [8] or PCT [18] to predict prognosis of IE. Most of the proposed risk factors for poor prognosis in IE had to be rejected over time. The remaining factors that are indicative for a poor prognosis comprise a) development of complications, e.g. heart failure, septic emboli or persistent bacteremia, b) indicators of excessive valve destruction, e.g. vegetation size or perivalvular abscess formation, c) concomitant diseases, e.g. diabetes mellitus, d) the causative organism and e) increasing age [19]. Older patients are less likely to undergo surgery for IE because of comorbidities, even if surgery is indicated. Excessive valve destruction makes surgery more often necessary.

We were the first to show that PCT levels on admission of patients with death or serious complications were significantly higher compared to those in event-free survivors. The accuracy of PCT to predict death or serious complications at follow-up was superior to CRP and WBC. A PCT cut-off > 0.5 ng/mL was the best predictor of adverse outcome. If our findings are confirmed in future prospective intervention studies, PCT might become part of a tool for risk assessment in IE, with PCT levels ≤ 0.5 ng/mL indicating low-risk patients. In high-risk patients with PCT levels > 0.5 ng/mL, increased attention to complications of IE and closer medical follow-up may be indicated. This cut-off for PCT > 0.5 ng/mL is similar to the PCT cut-off used for the diagnosis of sepsis, which Kocazeybek et al. also used in their study on serial measurements of PCT [18] without further statistical evaluation. This seems plausible because IE is an intracardiac infection with continuous bacteremia. As a result PCT levels in IE are higher than in other infections like e.g. lower respiratory tract infections, where a lower PCT cut-off of 0.25 ng/mL is applied whereas they are comparable to sepsis, where a PCT cut-off of 0.5 ng/mL is used [20].

As a marker for poor outcome in cardiac diseases, B-type natriuretic peptide has been extensively studied. Its initial levels do show association to clinical outcome in infective IE, but adequate analysis with respect to diagnostic cut-offs and diagnostic accuracy is lacking [21,22].

A tool to predict individual prognosis in a severe disease such as IE would allow for more rationale resource allocation in the early and the late stages of treatment. Initially, allocation to different setting of care such as intensive, intermediate and standard care wards might be more straightforward.

Over the past decade, PCT has gained importance in the diagnosis of severe systemic infection and sepsis [23]. In lower respiratory tract infections and exacerbation of chronic obstructive lung disease, it is used to guide antibiotic therapy [24]. We hypothesized that PCT might be useful to predict microbiological etiology of IE, which would be helpful for the choice of adequate antibiotic therapy. One of the most powerful indicators of poor clinical outcome is staphylococcal IE, which is associated with more frequent rapid course of the disease and more complications. This was also true in our study cohort, where 15 out of 16 patients (94%) with *Staphylococcus aureus* IE experienced serious complications. The results of blood cultures are normally not available at the time of initiation of antibiotic therapy. If blood cultures become positive the microbiological results are usually available within 24 to 48 hours. According to the AHA guidelines, the choice of appropriate antibiotic therapy for IE depends on the causative microorganism. Jereb et al. found highest PCT levels in patients with *Staphylococcus aureus* endocarditis [12]. We also found that patients with IE due to *Staphylococcus aureus* were more likely to show higher values of PCT, CRP and WBC. In our study, the odds ratio for patients with PCT > 0.5 ng /mL was 12.8 for the prediction of *Staphylococcus aureus* IE. However, inflammatory markers did not allow an individual prediction of microbial etiology of IE. As a consequence an empiric antibiotic therapy for IE has to be started irrespective of the levels of the inflammatory markers. Antibiotic therapy should be adapted after blood culture results are available.

In lower respiratory tract infections there are several trials that clearly demonstrated that antibiotic therapy can be guided by PCT measurements [25-27]. This results in a reduction of antibiotic consumption compared to a standard therapy without PCT guidance. In IE there is no such trial with a PCT algorithm for guidance of antibiotic therapy. Taking into account the high risk of recurrence of IE, such a PCT algorithm should be different from the one used for lower respiratory tract infections. In patients with a high probability of IE, antibiotic therapy would not be withheld from any patient, irrespective of the PCT value. However, one might speculate that it might be possible to stop antibiotic therapy successfully, if PCT values have fallen for some weeks below a specific cut-off value.

The present study has some limitations. Despite intensive microbiological diagnostics, the etiology remained unknown in 14%. This problem is well known from other IE studies [8-10]. We did not perform serial PCT measurements. Therefore we were not able to correlate PCT kinetics to clinical outcome variables. Also, the sample size did not allow for reliable multivariate analysis. A prospective multicentre study is needed to address these limitations.

## Conclusions

Appropriate biomarkers for predicting prognosis in IE would be helpful for optimal management of this disease and rational resource allocation. This study shows that PCT is a possible predictor of poor prognosis. Measurement of PCT, CRP, and WBC however do not allow an individual prediction of microbial etiology of IE.

PCT might be a valuable tool helping clinicians – in combination with complimentary clinical data - to assess a patient’s risk profile and to improve therapeutic decision making.

## Competing interests

The authors declare that they do not have any financial or non-financial competing interests.

## Authors’ contributions

CGC, NM and SK conceived the concept and design of the study. Data was gathered by CGC and KP. CGC and SK performed the data analysis. CGC, DAF and SK wrote the manuscript. All authors read and approved the final manuscript.

## Pre-publication history

The pre-publication history for this paper can be accessed here:

http://www.biomedcentral.com/1471-2334/13/272/prepub
